# Supplementary material for: PRMT5-mediated methylation of STAT3 is required for lung cancer stem cell maintenance and tumour growth
Source: Commun Biol. 2024 May 17;7:593. doi: 10.1038/s42003-024-06290-7 (PMC11101626; doi:10.1038/s42003-024-06290-7)
Supplement: Supplementary file 2 — Supplementary Information [file 42003_2024_6290_MOESM2_ESM.pdf]

**Supplementary Information**

# PRMT5-mediated methylation of STAT3 is required for lung cancer stem cell maintenance and tumour growth

**Yoshinori Abe, Takumi Sano, Naoki Otsuka, Masashi Ogawa, and Nobuyuki Tanaka**

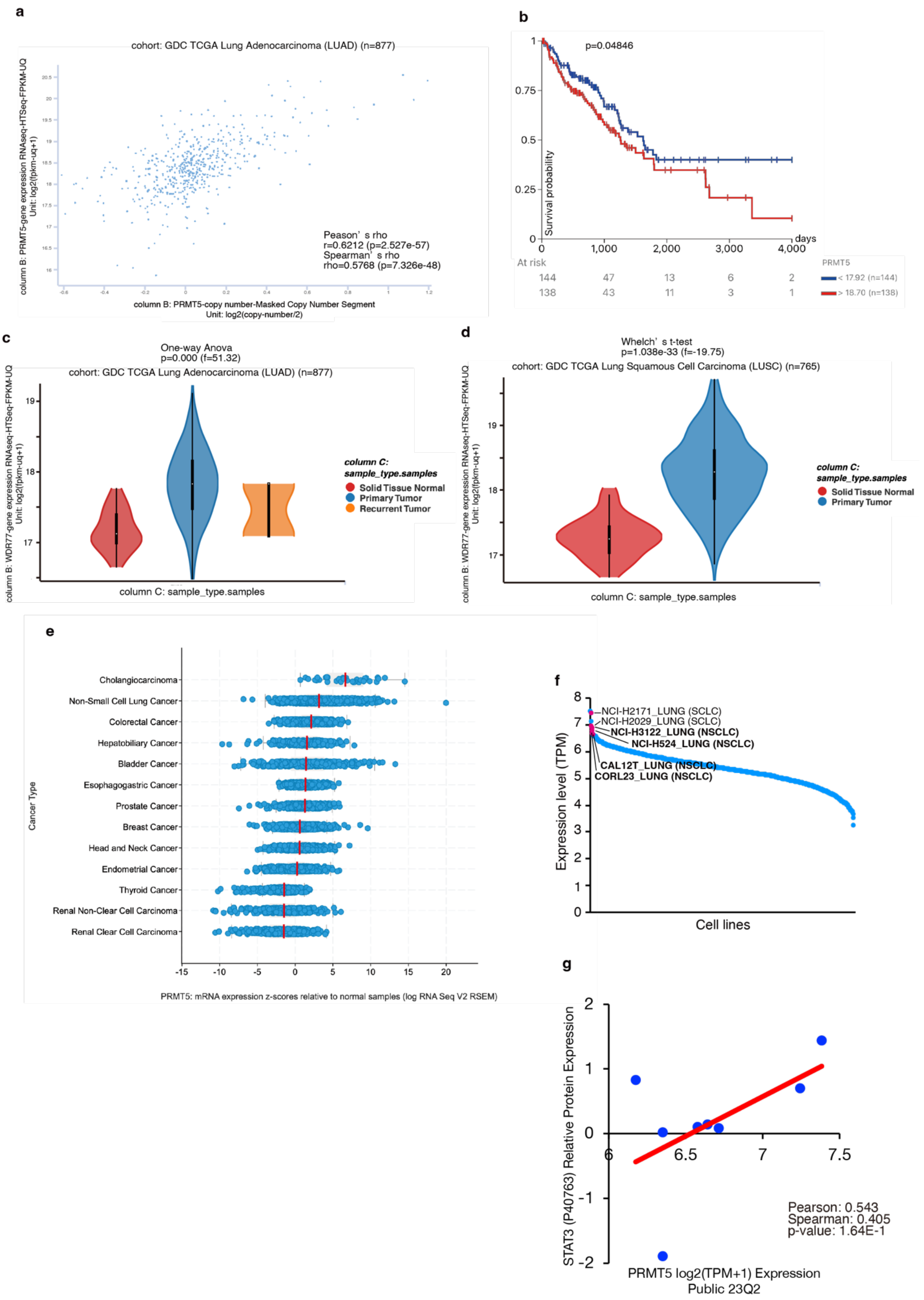
a
cohort: GDC TCGA Lung Adenocarcinoma (LUAD) (n=877)
column B: PRMT5-gene expression RNAseq-HTSeq-FPKM-UQ
Unit: log2(fpkm-uq+1)
Peason' s rho
r=0.6212 (p=2.527e-57)
Spearman' s rho
rho=0.5768 (p=7.326e-48)
column B: PRMT5-copy number-Masked Copy Number Segment
Unit: log2(copy-number/2)
b
p=0.04846
Survival probability
days
At risk
144 47 13 6 2
138 43 11 3 1
PRMT5
< 17.92 (n=144)
> 18.70 (n=138)
c
One-way Anova
p=0.000 (f=51.32)
cohort: GDC TCGA Lung Adenocarcinoma (LUAD) (n=877)
column B: WDR77-gene expression RNAseq-HTSeq-FPKM-UQ
Unit: log2(fpkm-uq+1)
column C:
sample_type.samples
Solid Tissue Normal
Primary Tumor
Recurrent Tumor
column C: sample_type.samples
d
Whelch' s t-test
p=1.038e-33 (f=-19.75)
cohort: GDC TCGA Lung Squamous Cell Carcinoma (LUSC) (n=765)
column B: WDR77-gene expression RNAseq-HTSeq-FPKM-UQ
Unit: log2(fpkm-uq+1)
column C:
sample_type.samples
Solid Tissue Normal
Primary Tumor
column C: sample_type.samples
e
Cancer Type
Cholangiocarcinoma
Non-Small Cell Lung Cancer
Colorectal Cancer
Hepatobiliary Cancer
Bladder Cancer
Esophagogastric Cancer
Prostate Cancer
Breast Cancer
Head and Neck Cancer
Endometrial Cancer
Thyroid Cancer
Renal Non-Clear Cell Carcinoma
Renal Clear Cell Carcinoma
PRMT5: mRNA expression z-scores relative to normal samples (log RNA Seq V2 RSEM)
f
Expression level (TPM)
NCI-H2171_LUNG (SCLC)
NCI-H2029_LUNG (SCLC)
NCI-H3122_LUNG (NSCLC)
NCI-H524_LUNG (NSCLC)
CAL12T_LUNG (NSCLC)
CORL23_LUNG (NSCLC)
Cell lines
g
STAT3 (P40763) Relative Protein Expression
Pearson: 0.543
Spearman: 0.405
p-value: 1.64E-1
PRMT5 log2(TPM+1) Expression
Public 23Q2

**Supplementary Figure 1**

**a** Correlation between PRMT5 copy number and *PRMT5* expression level in lung adenocarcinoma. **b** Higher *PRMT5* expression levels are associated with worse overall survival. All data were obtained from The Cancer Genome Atlas (TCGA) and analysed using the UCSC Xena browser (https://xena.ucsc.edu). **c**, **d** *MEP50* expression is enhanced in **c)** lung adenocarcinoma and **d)** lung squamous cell carcinoma. RNA-seq data from TCGA were analysed on the UCSC Xena browser. **e** Ranking of *PRMT5* expression levels in 13 cancer types from TCGA Pan-Cancer Atlas Studies (10,967 samples from 10,953 patients). RNA-seq data were analysed on cBioPortal, and *PRMT5* expression was ranked by z-score. **f** Ranking of *PRMT5* expression in 1,019 cancer cell lines. RNA-seq data were obtained from the Cancer Cell Line Encyclopedia. **g** Correlation between STAT3 protein level and *PRMT5* mRNA level in eight NSCLC cell lines. The data were obtained from the Cancer Cell Line Encyclopedia and obtained expression data were analysed using Data Explorer at the depmap portal (https://depmap.org/portal/).

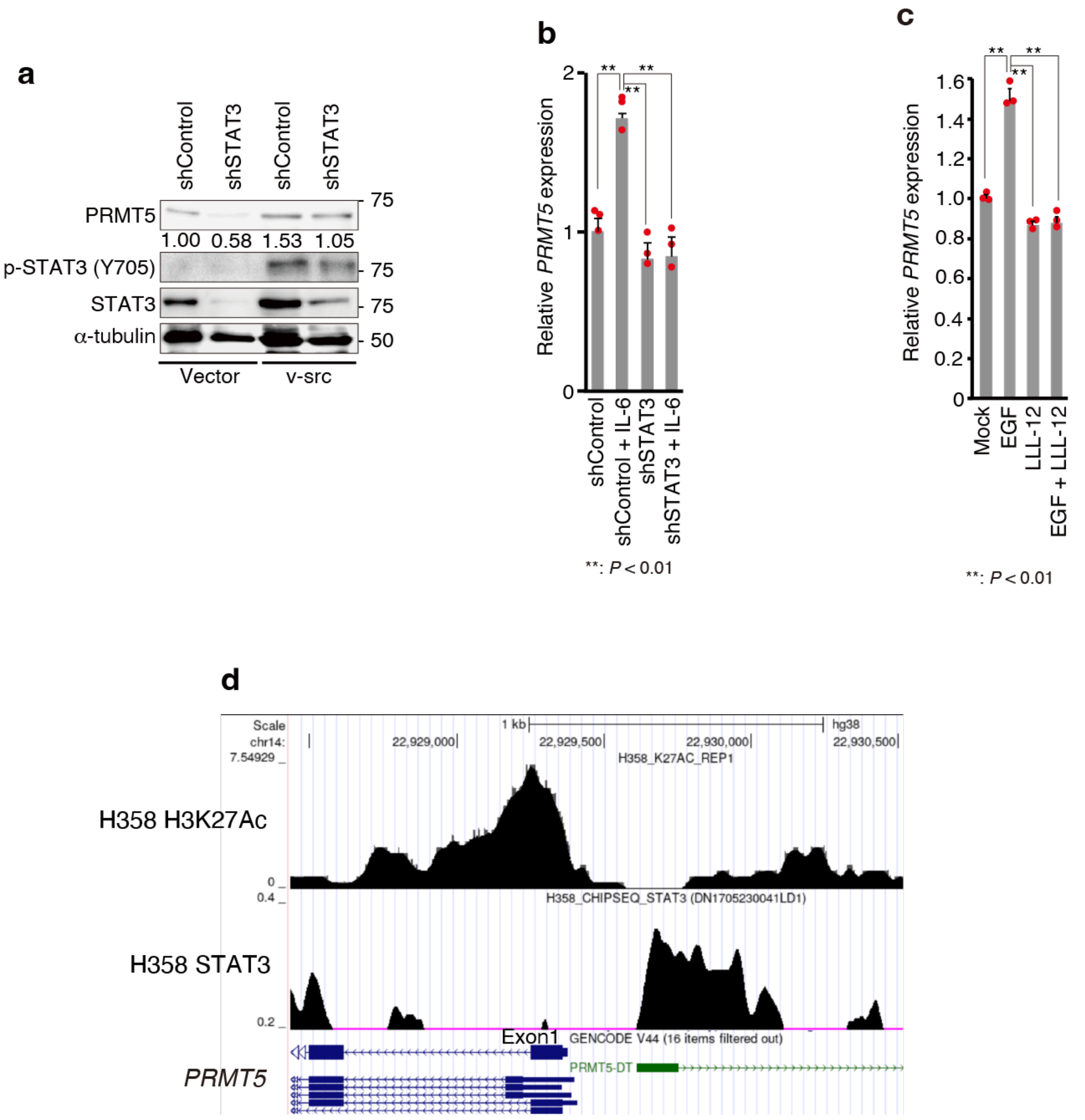


**Supplementary Figure 2**

**a** v-SRC-mediated STAT3 activation enhances PRMT5 expression in NIH3T3 cells. v-SRC was stably expressed by a recombinant retrovirus. STAT3 activation was assessed by western blot analysis using an anti-phospho-STAT3 (Y705) antibody (CST; 9138). Protein expression levels were determined by western blot analysis using antibodies for the indicated proteins [PRMT5: Abcam (ab109451), STAT3: CST (12640), α-tubulin: Sigma-Aldrich (T6199)]. PRMT5 expression levels were quantified with normalization to the α-tubulin expression intensity. **b, c** Recombinant interleukin (IL)-6 **b**) or epidermal growth factor (EGF)-mediated **c**) STAT3 activation enhances *PRMT5* expression in NIH3T3 cells. Cells were treated with 50 ng/ml recombinant IL-6 and 50 ng/ml soluble

IL-6 receptor (sIL-6R) or 20 ng/ml recombinant EGF for 3 h before harvesting. *PRMT5* expression levels were analysed by qPCR. Results are shown as the mean ± standard deviation (SD) from three experiments. Tukey's honestly significant difference test was applied for statistical comparisons. **d** The UCSC Genome Browser (http://genome.ucsc.edu/index.html) results show the locations of STAT3 ChIP-Seq and acetylated histone H3 at Lys27 (H3K27Ac) ChIP-Seq signals on the PRMT5 locus in H358, which harbors the KRAS mutation (KRAS G12C). Source data are provided in Supplementary Data 2. Full immunoblot images are shown in Supplementary Figure 8.

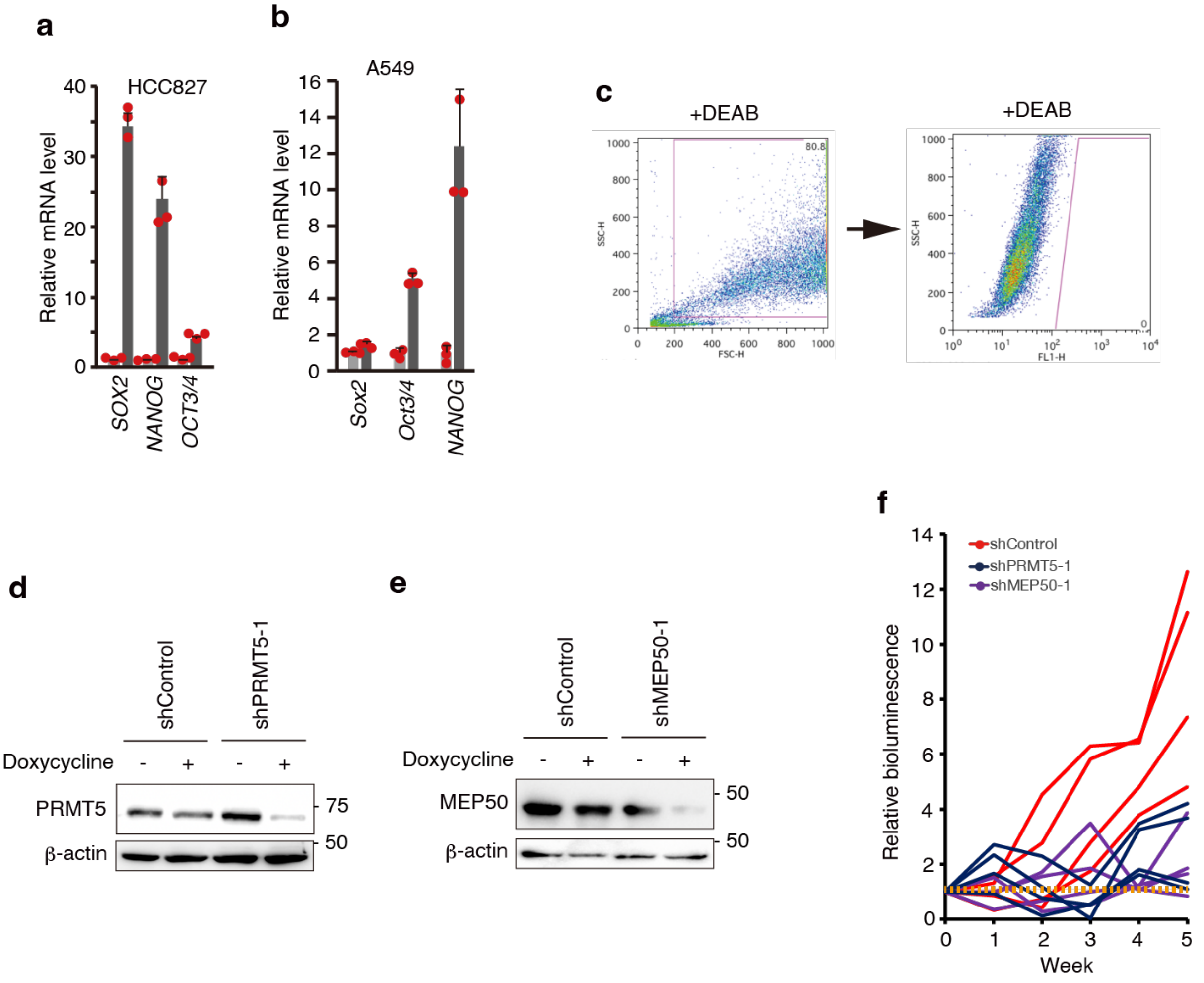


**Supplementary Figure 3**

**a, b** qPCR analysis of cancer stem cell marker genes in adherent cells and tumour spheres in **a)** HCC827 and **b)** A549 cells. Results are shown as the mean ± standard deviation (SD) from three experiments. **c** Gating strategy for flow cytometry using parental HCC827 cells. Debris and non-viable cells were excluded in the Side Scatter (SSC) versus Forward Scatter (FSC) plot (left). The gating of cells having high aldehyde dehydrogenase (ALDH) activity was based on the SSC versus FL1 (FITC) plot of 4-diethylaminobenzaldehyde (DEAB)-treated cells (right). **d, e** Decrease of PRMT5 or MEP50 protein level by inducible expression of shRNA for PRMT5 or MEP50. Doxycycline was applied at 50 ng/ml for 72 h before harvesting. Protein expression levels were detected by western blot analysis using antibodies for the indicated proteins (PRMT5: Abcam ab109451, MEP50: Abcam ab154190). **f** Relative tumor volume of individual mice shown in Figure 3i. The Orange dashed line denotes the baseline (Relative bioluminescence=1). Source data are provided as Supplementary Data 2. Full immunoblot images are shown in Supplementary Figure 8.

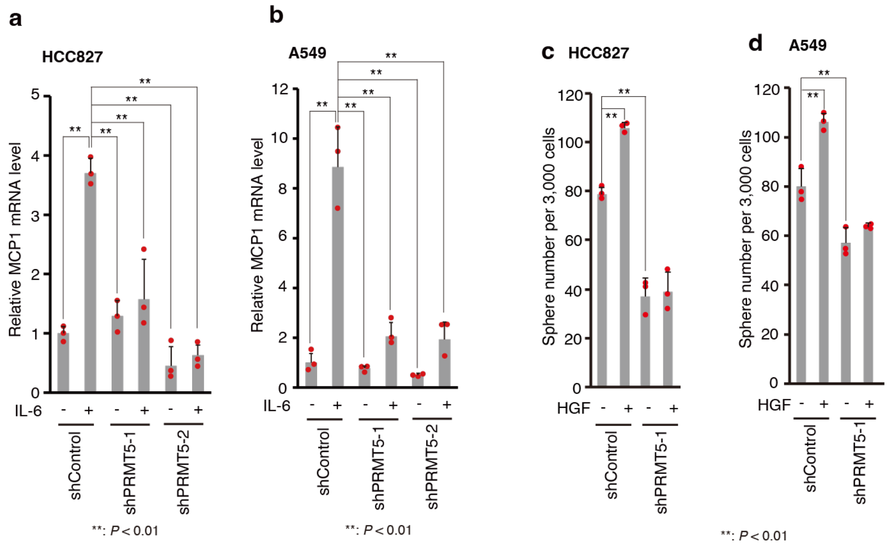


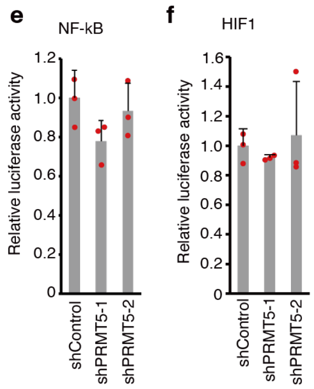


**g**

| R site | Peptides | Prediction score |
|---|---|---|
| 214 | QMLTALDQMRRSIVSELAG | 0.736543 |
| 215 | MLTALDQMRRSIVSELAGL | 0.640023 |
| **379** | **KDSGDVAALRGSRKFNILG** | **0.975005** |
| 414 | SAEFKHLTLREQRCGNGGR | 0.731902 |
| 417 | FKHLTLREQRCGNGGRANC | 0.778671 |
| **423** | **REQRCGNGGRANCDASLIV** | **0.821318** |
| **518** | **SWQFSSTTKRGLSIEQLTT** | **0.843786** |
| 593 | YIMGFISKERERAILSTKP | 0.51627 |
| 595 | MGFISKERERAILSTKPPG | 0.68447 |
| **609** | **TKPPGTFLLRFSESSKEGG** | **0.917535** |

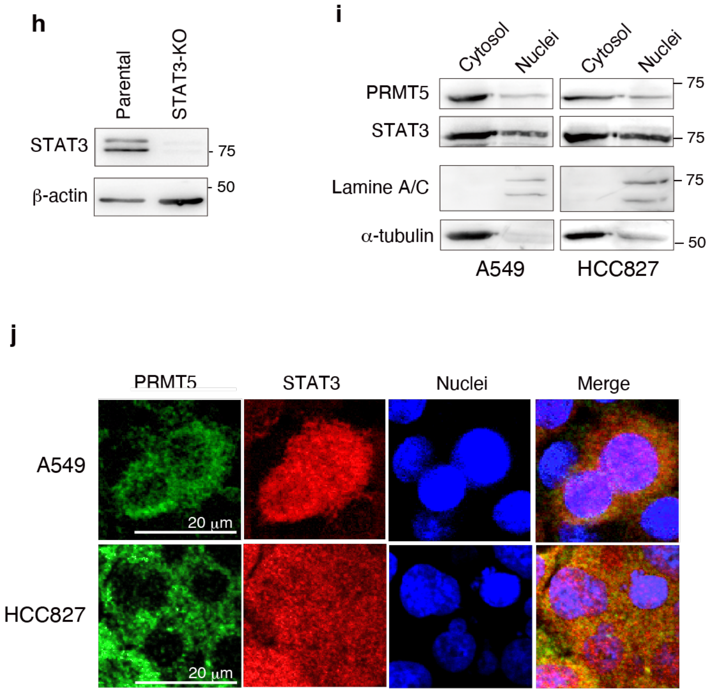

**Supplementary Figure 4**

**a, b** Knockdown of PRMT5 suppresses IL-6-STAT3 axis-mediated *MCP1* expression in **a)** HCC827 and **b)** A549 cells. 50 ng/ml recombinant IL-6 and 50 ng/ml soluble IL-6 receptor (sIL-6R) were treated for 3 h before harvesting. *MCP1* expression levels were assessed by qPCR. The results are shown as the mean ± standard deviation (SD) from three experiments. Tukey's honestly significant difference test was applied for statistical comparisons. **c, d** Sphere numbers with hepatocyte growth factor (HGF) treatment in shControl- and shPRMT5-expressing **c)** HCC827 and **d)** A549 cells. Cells were treated with 50 ng/ml recombinant HGF for 7 days, after which sphere numbers were counted. The results are shown as the mean ± standard deviation (SD) from three experiments. Tukey's honestly significant difference test was applied for statistical comparisons. **e, f** Knockdown of PRMT5 did not affect **e)** NF-κB or **f)** HIF1 transcriptional activity. NF-κB or HIF1 transcriptional activity was assessed by luciferase assays using an NF-κB or HIF1 reporter plasmid in A549 cells. Results are shown as the mean ± SD from three experiments. **g** The arginine methylation sites in STAT3 were predicted by PRmePRed (http://bioinfo.icgeb.res.in/PRmePRed/index.html). **h** STAT3 expression in STAT3-knockout BEAS2B cells. STAT3 knockout was performed using the CRISPR–Cas9 system. STAT3 protein levels were detected by western blot analysis using an anti-STAT3 antibody (CST; 12640). **i** PRMT5 is abundant in the cytoplasm. Cells were lysed and separated into cytosolic and nuclear fractions. Nuclear or cytosolic PRMT5 and STAT3 were detected by western blotting using antibodies against PRMT5 (Abcam; ab109451) and STAT3 (CST; 12640). Lamin A/C, a nuclear marker, was detected by western blotting using an anti-Lamin A/C antibody (Santa Cruz; sc-7293), and α-tubulin, a cytoplasmic marker, was detected by western blotting using an α-tubulin antibody (Sigma-Aldrich; T6199). **j** Immunofluorescence images of PRMT5 and STAT3 localization in A549 cells. PRMT5 was detected using a PRMT5 antibody (Santa Cruz; sc-376937), and STAT3 was detected using an STAT3 antibody (CST; 12640). Nuclei were stained using Hoechst33342. Representative results in a–f were from two independent results. Bar, 20 μm. Source data are provided in Supplementary Data 2. Full immunoblot images are shown in Supplementary Figure 8.

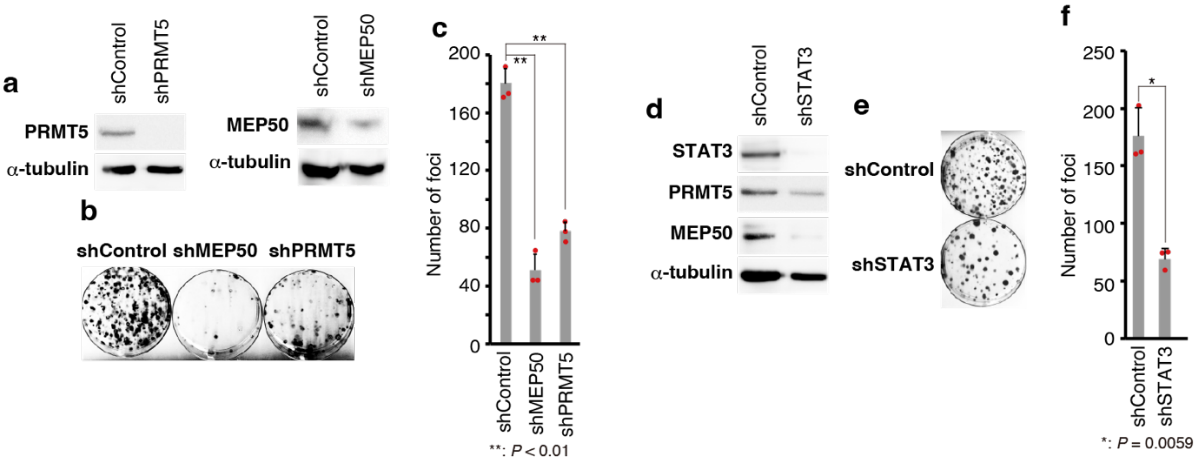


**Supplementary Figure 5**

**a–f** A quantitative focus formation assay was performed by plating v-*src*- and short hairpin RNA (shRNA) (shPRMT5 **(a–c)**, shMEP50 **(a–c)**, or shSTAT3 **(d–f)**)-expressing *p53* null mouse embryonic fibroblasts (MEFs) at a density of $1 \times 10^3$ cells per 10 cm dish. After 14 days of culture, cells were fixed with methanol and stained with crystal violet. **a, d** Western blot analysis of endogenous protein levels (a: shPRMT5 and shMEP50, d: shSTAT3). shRNAs for PRMT5, MEP50, or STAT3 were stably expressed by recombinant retrovirus. **b, e** Representative plate image (b: shPRMT5 and shMEP50, e: shSTAT3). **c, f** Quantification of foci (c: shPRMT5 and shMEP50, f: shSTAT3). Representative results from two independent experiments are shown in c. Source data are provided in Supplementary Data 2. Full immunoblot images are shown in Supplementary Figure 8.

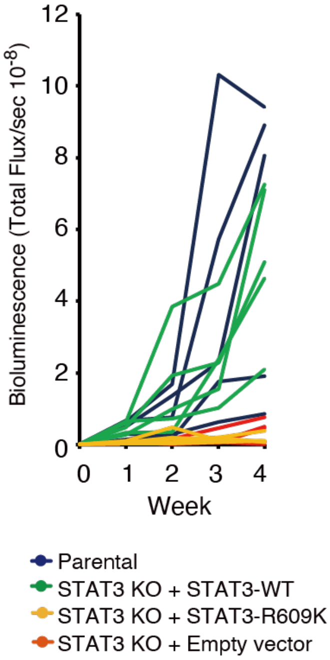


**Supplementary Figure 6**

Tumor volume of individual mice shown in Figure 5f.

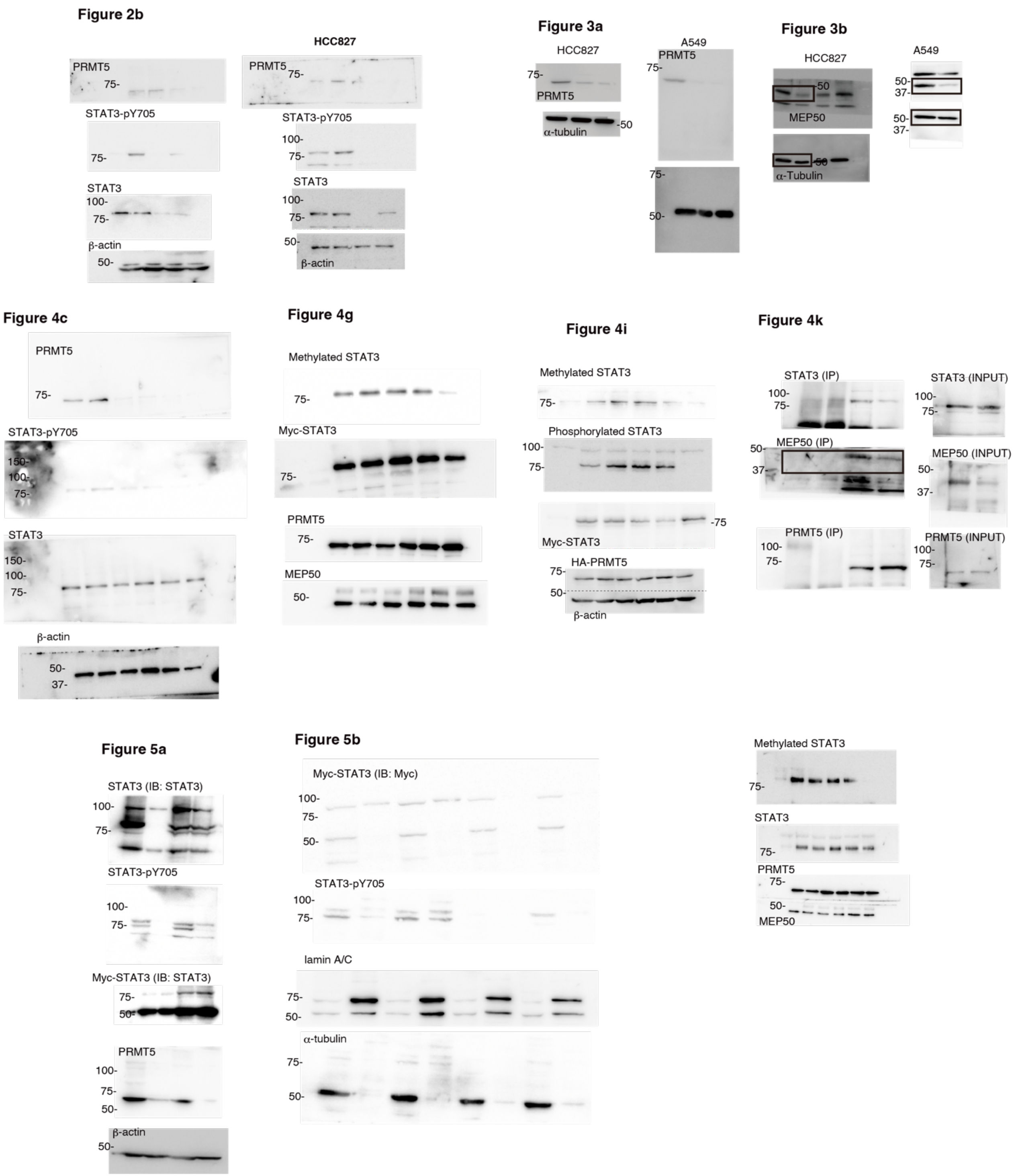


## Supplementary Figure 7

The original blots shown in Figures 2–5.

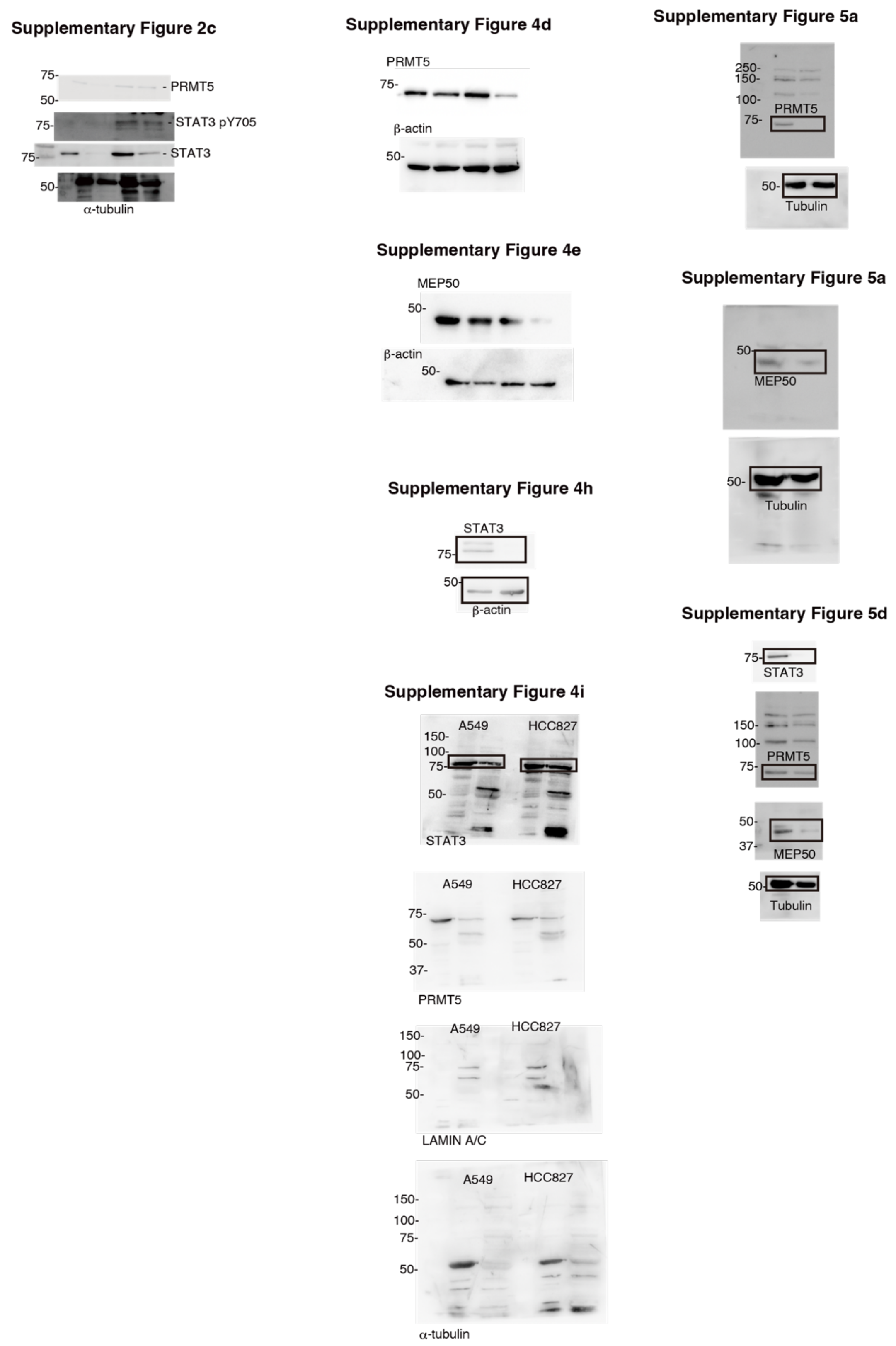


**Supplementary Figure 8**

The original blots shown in Supplementary Figures 2, 4 and 5.

**Supplementary Table 1: Antibodies used in this study.**

| Antibody (clone) | Manufacturer (Catalog number) | Usage: Dilution |
|---|---|---|
| PRMT5 (EPR5772) | Abcam (ab109451) | IB: 1:2000<br>IP: 1 μl |
| PRMT5 (A-11) | Santa Cruz (Santa Cruz, CA, USA; sc-376937) | IF: 1:200 |
| MEP50 (EPR10708(B)) | Abcam (Cambridge, UK; ab154190) | IB: 1:1000 |
| MEP50 (3F10) | Abnova (Taipei, Taiwan; H00079084-M01) | IB: 1:1000 |
| STAT3 (D3Z2G) | Cell Signaling Technology (12640) | IB: 1:1000<br>IF: 1:500<br>Ch: 1:50 |
| STAT3 (124H6) | Cell Signaling Technology (9139) | IB: 1:1000 |
| Phospho-STAT3-Tyr705 (D3A7) | Cell Signaling Technology (9138) | IB: 1:1000 |
| Phospho-STAT3-Tyr705 (EPR23958-52) | Abcam (ab267373) | IB: 1:1000 |
| Symmetric di-methyl arginine (SYM11) | Merck Millipore (07-413) | IB: 1:700 |
| LAMIN A/C (346) | Santa Cruz (sc-7293) | IB: 1:1000 |
| HA-tag (16B12) | Abcam (ab130275) | IB: 1:1000 |
| Myc-tag (9E10) | Abcam (ab32) | IB: 1:1000 |
| Myc-tag | GeneTex (Irvine, CA, USA; GTX115046) | IB: 1:1000 |
| FLAG tag | Sigma-Aldrich (SAB4301135) | IB: 1:1000 |
| His tag | Proteintech (Rosemont, IL, USA; 10001-0-AP) | IB: 1:1000 |
| α-tubulin (DM1A) | Sigma-Aldrich (T6199) | IB: 1:2000 |
| β-actin (AC-74) | Sigma-Aldrich (A2228) | IB: 1:2000 |
| Rabbit IgG XP Isotype control | Cell Signaling Technology (3900) | Ch: 1:50 |
| Anti-Rabbit IgG, HRP-Linked F (ab') $_2$ Fragment Donkey (secondary Ab) | Cytiva (NA9340) | IB: 1:5000 |
| Anti-Mouse IgG, HRP-Linked F (ab') $_2$ Fragment Sheep (secondary Ab) | Cytiva (NA9310) | IB: 1:5000 |
| Alexa-488-conjugated anti-Mouse IgG (secondary Ab) | ThermoFisher Scientific (A11017) | IF: 1:500 |
| Alexa-568-conjugated anti-Rabbit IgG (secondary Ab) | ThermoFisher Scientific (A11011) | IF: 1:500 |

IB: Immunoblot, IP: Immunoprecipitation, Ch: Chromatin Immunoprecipitation (ChIP) assay, IF: Immunofluorescence

**Supplementary Table 2: Short hairpin RNA (shRNA) sequences used for RNA interference (RNAi) experiments.**

| shRNA | Sequence |
|---|---|
| Human shPRMT5-1 | GGAATCTCAGACATATGAAGTTTCAA<br>GAGAACTTCATATGTCTGAGATTCC |
| Human shPRMT5-2 | GCTATTGCACCTTGGAATTTCTTCAA<br>GAGAGAAATTCCAAGGTGCAATAGC |
| Mouse shPRMT5-1 | GACTCTAGTATCAAGGAATTCAA<br>GAGATTCCTTGATACTAGAGTCC |
| Human shMEP50-1 | GGACTCTGTGTTTCTTTCATTCAA<br>GAGATGAAAGAAACACAGAGTCC |
| Mouse shMEP50-1 | GCATGCATGGAACGTCAATTTCA<br>AGAGAATTGACGTTCCATGCATGC |
| Human shSTAT3-1 | GCCCGTCAACAAATTAAGAACTC<br>GAGTTCTTAATTTGTTGACGGG |
| Human shSTAT3-2 | GCCTCTCTGCAGAATTCAACTCGA<br>GTTGAATTCTGCAGAGAGGC |
| Human shSTAT3-3 | GGTACAACATGCTGACCAATTCAA<br>GAGATTGGTCAGCATGTTGTACC |
| Human shSTAT3-4 | GCTGAACAACATGTCATTTCTCGA<br>GAAATGACATGTTGTTCAGC |
| Mouse shSTAT3 | GCACAACCTTCGAAGAATCTTCAAGA<br>GAGATTCTTCGAAGGTTGTGCTTTTTA |
| Control shRNA<br>(shControl) | ATCTCGCTTGGGCGAGAGTAAGTTCAA<br>GAGACTTACTCTCGCCCAAGCGAGAT |

Underlined sequences indicate the hairpin loop sequence.

**Supplementary Table 3: Guide RNAs for CRISPR–Cas9-mediated gene knockout.**

| Gene | Sequence | Exon / Intron | Strand |
|---|---|---|---|
| STAT3 (#1) | TCCAGTTCACTACTAAAGTC | Exon | - |
| STAT3 (#2) | TCAAACACTTGGTATGTGGG | Intron | - |

STAT3 (#1) was used for STAT3 knockout in BEAS2B cells and STAT3 (#2) was used for STAT3 knockout in HCC827 cells.

**Supplementary Table 4: PCR primer list for generation of mutant STAT3.**

| STAT3 mutant | Primer |
|---|---|
| STAT3-R379K | Sense – CGTTGCAGCTCTCAAAGGATCCCGGAAAT<br>Antisense – ATTTCCGGGATCCTTTGAGAGCTGCAACG |
| STAT3-R423K | Sense – TGGGAATGGGGGCAAAGCCAATTGTGATG<br>Antisense – CATCACAATTGGCTTTGCCCCCATTCCCA |
| STAT3-R518K | Sense – CTCCACCACCAAGAAAGGACTGAGCATCG<br>Antisense – CGATGCTCAGTCCTTTCTTGGTGGTGGAG |
| STAT3-R609K | Sense – CACCTTCCTGCTAAAATTCAGTGAAAGCA<br>Antisense – TGCTTTCACTGAATTTTAGCAGGAAGGTG |

**Supplementary Table 5: TaqMan probes used in this study.**

| Gene Symbol | Assay ID |
|---|---|
| PRMT5 | Hs01047345_g1 |
| MCP-1 | Hs00234140_m1 |
| β-actin | Hs01060665_g1 |

**Supplementary Table 6: Primer list for ChIP-qPCR.**

| Region | Primer sequence |
|---|---|
| -1231 – -1224 | Forward: GCCTGCTACCACGTTTAGGA<br>Reverse: GAATGAAAGCCGGAAAAGCG |
| -1139 – -1130 | Forward: GGCTTTCATTCTTTTGGGGC<br>Reverse: GTTGGATGGAGGTCGCTAGA |
| -959 – -951 | Forward: TCTAGCGACCTCCATCCAAC<br>Reverse: CCTGAGAAAGCAAAGTAGGGG |
| -627 – -620 | Forward: CCTCCCTATGTCTTTCCTTGC<br>Reverse: AAACGCCTAAACACACACAC |
| -540 – -532 | Forward: TGTGTGTGTGTTTAGGCGTTT |

| | |
|---|---|
| -500 – -492 | Reverse: AACTGGGTTAATGGCTGGTG |
| -375 – -366 | Forward: AGGGTAGTTACGGGAAAAGCT<br>Reverse: CCTTTCCTTTCGCAGCTTGA |
| -267 – -260 | Forward: TGCATAACCCAACCGACTCA<br>Reverse: GGGGTTCCTCGCAGCTTC |
