# Supplementary material for: PRMT5-mediated methylation of STAT3 is required for lung cancer stem cell maintenance and tumour growth
Source: Commun Biol. 2024 May 17;7:593. doi: 10.1038/s42003-024-06290-7 (PMC11101626; doi:10.1038/s42003-024-06290-7)
Supplement: Supplementary file 3 — Description of Additional Supplementary Filese [file 42003_2024_6290_MOESM3_ESM.pdf]

Description of Additional Supplementary Files

File name: Supplementary Data 1
Description: The source data behind the graph in the main figures.

File name: Supplementary Data 2
Description: The source data behind the graph in the supplementary figures.
